# Resveratrol ameliorates liver fibrosis induced by nonpathogenic *Staphylococcus* in BALB/c mice through inhibiting its growth

**DOI:** 10.1186/s10020-022-00463-y

**Published:** 2022-05-04

**Authors:** Zhiqin Li, Jianxia Dong, Meng Wang, Jingya Yan, Yushu Hu, Yang Liu, Yajie Pan, Hua Li

**Affiliations:** 1 Department of Infectious Diseases, The First Affiliated Hospital of Zhengzhou University, No. 1, Jianshe East Road, Zhengzhou, 450001 Henan Province China; 2 Department of Gastroenterology, The First Affiliated Hospital of Zhengzhou University, No. 1, Jianshe East Road, Zhengzhou, 450001 Henan Province China

**Keywords:** Resveratrol, Liver fibrosis, *Staphylococcus_xylosus* and *Staphylococcus_lentus*, Bacterial translocation, Intestinal permeability

## Abstract

**Background:**

The altered gut microbiota is implicated in the pathogenesis of liver fibrosis. Resveratrol is a candidate for the treatment of liver fibrosis, which could ameliorate the dysregulation of gut microbiota in mice. This study aimed to clarify the role and mechanism of resveratrol in gut microbiota during liver fibrosis.

**Methods:**

A mouse model of liver fibrosis induced by CCl_4_ was conducted to assess the effect of resveratrol on liver fibrosis. The changes of gut microbiota in liver fibrotic mice after resveratrol intervention were assessed using 16S ribosomal RNA sequencing. The mechanism of the gut microbiota dysregulation in liver fibrosis was investigated by Sirius red staining, immunohistochemical assay, bacterial translocation (BT), EUB338 fluorescence in situ hybridization, immunofluorescence, trans-epithelial electrical resistance analysis and paracellular permeability analysis.

**Results:**

Resveratrol relieved CCl_4_-induced liver fibrosis. Besides, resveratrol restrained the gut microbiota *Staphylococcus_lentus* and *Staphylococcus_xylosus* in the liver fibrotic mice, and the *Staphylococcus_xylosus* and *Staphylococcus_lentus* facilitated the occurrence of BT and the cultures of them enhanced the permeability of intestine. The in vivo assay corroborated that the excessive *Staphylococcus_xylosus* and *Staphylococcus_lentus* canceled the protecting effect of resveratrol on liver fibrosis, and *Staphylococcus_xylosus* or *Staphylococcus_lentus* alone had a limited impact on the liver injury of normal mice.

**Conclusion:**

Resveratrol ameliorated liver fibrosis by restraining the growth of *Staphylococcus_xylosus* and *Staphylococcus_lentus*.

**Supplementary Information:**

The online version contains supplementary material available at 10.1186/s10020-022-00463-y.

## Introduction

Liver fibrosis is the final adverse outcome of chronic liver injury induced by multiple factors, including nonalcoholic steatohepatitis, alcohol abuse, and metal poisoning (Campana and Iredale 2017). Although liver fibrosis is reversible, without timely intervention or treatment, liver fibrosis will gradually develop into liver cirrhosis with a high mortality rate (Lin et al. 2018; Smid 2020). Thus, revealing more potential mechanisms that induce liver fibrosis is of great significance for reducing liver fibrosis.

In the field of anatomy, the intestine and liver are closely related and the gut microbiota is a key factor that induces liver diseases (Yu and Schwabe 2017; Tripathi et al. 2018). Recent studies have shown that the changes in gut microbiota composition facilitate the CCl_4_-induced liver fibrosis (Gomez-Hurtado 2011; Zhao 2021). Bacterial translocation (BT) is the migration of bacteria or bacterial products from the intestinal tract to the mesenteric lymph nodes (MLNs) or extra-intestinal organs, and BT has been identified as an important pathway in the regulation of CCl_4_-induced liver fibrosis via gut microbiota (Gomez-Hurtado 2011; Chen, et al. 2019). Previous studies have shown that the increased intestinal permeability caused by intestinal barrier dysfunction facilitates the occurrence of BT (Cardinale et al. 2020; Ponziani et al. 2018), which in turn accelerates the development of liver fibrosis (Debes et al. 2016). Therefore, revealing the molecular mechanism of reducing intestinal permeability and inhibiting BT are expected to alleviate liver fibrosis.

Resveratrol, a phenolic substance isolated from veratrol, has attracted the attention of researchers because of its wide range of functions (Breuss et al. 2019; Malaguarnera 2019). Accumulating evidence suggests that resveratrol has a certain alleviating effect in liver fibrosis (Yu 2019; Abdu and Al-Bogami 2019). The possible regulating mechanisms of resveratrol’s inhibitory effects on chronic liver injury are varied, including anti-oxidative and anti-inflammatory effects, and bacterial translocation, et al. (Bereswill 2010; Wang et al. 2016; Zhu et al. 2020; Bujanda et al. 2008). What attracts our attention is that Qiao et al. have confirmed that resveratrol improves the dysregulation of gut microbiota in mice (Qiao et al. 2014). Importantly, Chen et al. have corroborated that administration of resveratrol ameliorates liver diseases by inducing a decrease in intestinal permeability and the occurrence of BT in NASH rat model for steatohepatitis (Chen et al. 2020). However, the molecular mechanism of resveratrol restraining BT by reducing intestinal permeability has not been fully elucidated.

In the preliminary work, we corroborated that administration of resveratrol ameliorated the liver fibrosis induced by CCl_4_ in mice. Moreover, resveratrol altered gut microbiota, in which *Staphylococcus_xylosus* and *Staphylococcus_lentus* facilitated the development of liver fibrosis in mice. Thus, we further investigated the possible mechanism of resveratrol affecting liver fibrosis through regulation of *Staphylococcus*. Our study may offer a novel view to the mechanism of gut microbiotic alteration in liver fibrosis.

## Materials and methods

### Construction of mouse liver fibrosis model

Fifteen Balb/c male mice (6–7 weeks old) were purchased from the Shanghai SLAC Laboratory Animal Co., Ltd (Shanghai, China) and randomly grouped as follows: Control (n = 5), CCl_4_ (n = 5) and CCl_4_ + resveratrol (n = 5). For the CCl_4_ group, the mice were intraperitoneally injected with 0.5 µL/g CCl_4_ (dissolved in olive oil) twice a week for 4 weeks. For the Control group, the mice were intraperitoneally injected with the same amount of olive oil twice a week for 4 weeks. For the CCl_4_ + resveratrol group, the mice were treated with 30 mg/kg resveratrol daily by gavage, following by intraperitoneally injection of 0.5 µL/g CCl_4_ twice a week for 4 weeks. For the overlapped day (the day mice received both resveratrol and CCl_4_ subjection), the interval time between resveratrol gavage and CCl_4_ induction was approximately 5 h. Four weeks immediately after above treatment, the mice were sacrificed under euthanasia condition and the tissue samples and blood samples were collected. All the animal experiments were approved by the Animal Care and Use Committee of the First Affiliated Hospital of Zhengzhou University and followed the principles outlined in the Declaration of Helsinki.

### Sirius red staining

The degree of liver fibrosis in mice was evaluated using Sirius red staining following the previously described method with minor changes (Chen et al. 2017). The liver tissues of mice were prepared into 5 μm sections, and then the Sirius red staining solution (Solarbio, Beijing, China) was applied to stain the above sections referring to the standard procedure of the manufacturer. The Image J software (National Institutes of Health, Bethesda, MD) was conducted to quantify the area of liver fibrosis in mice.

### Immunohistochemical analysis

The mouse liver tissues were fixed with 4% paraformaldehyde and prepared into 5 μm sections. The sections were incubated with 3% hydrogen peroxide for 15 min and then continued to incubate with 5% BSA for approximately 20 min. Next, the sections were incubated with anti-α-SMA (#19245, 1:500, Santa Cruz Biotechnology, USA) and anti-Collagen I (#72026, 1:200, Santa Cruz Biotechnology) overnight at 4 °C. The sections were further incubated with the specific secondary antibodies at room temperature for 1 h. Subsequently, the sections were treated with DAB chromogen (Abcam, Cambridge, UK) and re-stained with hematoxylin. All data were assessed using microscope (DS-U3, Nikon, Tokyo, Japan) and ImageJ software (National Institutes of Health, Bethesda, MD). A minimum of 5 representative fields per biological replicate were taken into analysis.

### Detection of the serum markers for liver fibrosis

Alanine aminotransferase (ALT), aspartate aminotransferase (AST), alkaline phosphatase (ALP), indirect bilirubin (IBil), total bilirubin (TBil), direct bilirubin (DBil) and hydroxyproline (Hyp) levels are common indicators for evaluating liver function (Elsayed Elgarawany et al. 2020; Ambade et al. 2019). After collecting the mouse blood samples, the contents of ALT (ab282882, Abcam, UK), AST (ab263882), ALP (ab285274) and Hyp (A030-3-1, Jiancheng, Nanjing, China) were measured by corresponding ELISA kits followed the instructions of the manufacturer. The levels of IBil, TBil and DBil were detected using a standard clinical automated analyser (SRL, Tokyo, Japan).

### Immunofluorescence assay

After the mouse colon tissues were harvested, the tissues were fixed with 4% paraformaldehyde and prepared into 5 μm sections. Followed by the sections blocked with 5% BSA, the sections were incubated with anti-zonula occludens-1 (ZO-1, #13663, 1:400, Santa Cruz Biotechnology) at 4 °C overnight. Next, the above sections were incubated with the specific secondary antibodies at room temperature for approximately 1 h and were stained with 4,6-diamidino-2-phenylindole (DAPI, Solarbio) for 10 min. For the Caco-2 monolayer cells, the cells were fixed by 4% paraformaldehyde and the non-specific sites were blocked with 10% FBS. The remaining methods were the same as above. Ultimately, the immunofluorescence signals were observed under a confocal microscope (BX51TF, Olympus, Japan).

### 16S rRNA sequencing and data processing

The genomic DNA from mouse feces was isolated using a PowerSoil DNA Isolation Kit (MoBio Laboratories, Carlsbad, CA, USA). Then, the Hot Master PCR mixture (5Prime, Gaithersburg, MD, USA) and specific primers targeting the 16S rRNA region V4 were conducted to amplify the 16S rRNA gene. Ultimately, the amplified product was sent to the biotech company (TSINGKE, Beijing, China) for the sequencing analysis. For the data processing, the diversity and composition of microbial communities were assessed using alpha-diversity and beta-diversity (Sims et al. 2019); and the genus-level differences were identified and the ternary plot analysis, and the relative abundance of species to investigate the gut microbiota of mice with obvious changes after CCl_4_ induction and resveratrol intervention.

### Bacteria source and culture

*Staphylococcus_lentus*, *Aerococcus_viridans* and *Staphylococcus_xylosus* were from American Type Culture Collection (ATCC, USA). *Staphylococcus_lentus*, *Aerococcus_viridans* and *Staphylococcus_xylosus* were cultured in the Tryptic Soy Agar (TSA, Thermo Fisher Scientific, Waltham, MA, USA) with 5% defibrinated sheep blood at 37 °C. After that, these bacteria were further cultured in the Tryptic Soy Broth (TSB, Thermo Fisher Scientific) at 37 °C.

### In vivo verification of the regulation of *Staphylococcus_lentus* and *Staphylococcus_xylosus* on liver fibrosis

To explore the impact of *Staphylococcus_lentus*, *Aerococcus_viridans* and *Staphylococcus_xylosus* on liver fibrosis in vivo, the pure cultures (10^9^ CFU) of gut microbiota were given to the mice in the control, CCl_4_ or CCl_4_ + resveratrol group by gavage, and then the liver fibrosis model of mice was induced by intraperitoneal injection of CCl_4_ (0.5 µL/g). Four weeks later, the mice were sacrificed and the tissue samples and blood samples were isolated. All the animals were approved by the Animal Care and Use Committee of the First Affiliated Hospital of Zhengzhou University.

### Construction of in vitro model of *Staphylococcus* biofilm

The cover glass was soaked in the TSB medium. After autoclaving, the bacteria solution (*Staphylococcus_xylosus* and *Staphylococcus_lentus*) of the activated TSB liquid medium was inoculated in the TSA medium and cultured at 37 °C overnight. A single colony was selected and inoculated in the TSB medium at 37 °C overnight for nearly 12 h, then diluted to OD_600_ of 0.4, and further diluted 100-fold. Then, 2 mL of the bacterial liquid was inoculated into the 24-well plates with the cover glass, mixed and sealed with parafilm. The biofilms were formed by continuous incubation in a shaker at 37 °C for nearly 3 days.

### Growth characteristics

The growth characteristics of *Staphylococcus_xylosus* and *Staphylococcus_lentus* were conducted as the previously described methods with minor modifications (Rong et al. 2019). After *Staphylococcus_xylosus* and *Staphylococcus_lentus* were treated with resveratrol with the minimum inhibitory concentration (MIC, 260 μg/mL), 1/2 MIC, 1/4 MIC, and 1/8 MIC, the cell growth was measured at 0, 1, 2, 3, 4, 5, 6, 7, 8, 9, 10 h using OD_600_ nm on a microplate reader (Multiskan MK3, Thermo Fisher Scientific).

Besides, after *Staphylococcus_xylosus* and *Staphylococcus_lentus* and their biofilms were treated with 1U of penicillin (PNC), the *Staphylococcus_xylosus* *Staphylococcus_lentus* biofilms were further treated with 1/8 MIC, and the cell growth was measured at 0, 1, 2, 3, 4, 5, 6, 7, 8, 9, 10 h using OD_600_ nm on a microplate reader (Multiskan MK3, Thermo Fisher Scientific).

### Determination of bacterial translocation

Bacterial translocation (BT) is generally considered as the presence of viable organisms in the MLN culture (Teltschik et al. 2012). Specifically, the MLNs were separated aseptically from the ileocecal zone. After grinding the separation, the homogenized MLNs (100 μL) were put in the MacConkey (Thermo Fisher Scientific), Mueller–Hinton (Thermo Fisher Scientific), and whole blood agar (Bio Merieux, Lyon, France) at 37 °C for approximately 2 days. Ultimately, the number of bacteria per gram was quantified using BT (CFU/g).

### Detection of lipopolysaccharide-binding protein (LBP) concentration

In accordance with standard reagent manufacturer procedures, we quantified the concentrations of LBP and Albumin in mouse blood samples using a lipopolysaccharide-binding protein (LBP) assay kit (Mlbio, Shanghai, China).

### EUB338 fluorescence in situ hybridization analysis

EUB338 fluorescence in situ hybridization (FISH) was carried out following the reported methods (Huang et al. 2020). The colon tissues were fixed with 4% paraformaldehyde and prepared into 5 μm sections. Then the sections were deparaffinized using xylene and rehydrated through an ethanol gradient (95%, 10 min; 90%, 10 min) to water. Subsequently, the sections were incubated with a universal bacterial probe directed against the 16S rRNA gene (EUB338: [Cy3]-GCTGCCTCCCGTAGGAGT-[AmC7 ~ Q + Cy3es]) at 60 °C for nearly 3 h. The NON-EUB 338 probe was a negative control probe to identify the non-specific binding of fluorochromes. The sections were then washed, and the sections were counter-stained with DAPI. All images were obtained under a confocal microscope (BX51TF, Olympus, Japan).

### Quantitative real-time PCR (qRT-PCR)

The colon tissues and Caco-2 cells were harvested, and TRIzol reagent (Solarbio, Beijing, China) was applied to isolate the total RNA. The quantity and and quality of RNA were evaluated by a Nanodrop (Thermo Scientific) and limited the A260/A280 ratios from 1.9 to 2.1. Then, RNA was reversely transcribed into cDNA using the SuperScript IV First-Strand Synthesis System (Thermo Fisher Scientific). The cDNA was subjected to perform qRT-PCR detection on the Bio-Rad CFX96™ Real-Time System with SYBR Green I PCR mix (Thermo Fisher Scientific). GAPDH was used as an internal reference and the relative expression of genes was calculated using the 2^−ΔΔCt^ method. The primers of ZO-1, occludin, ACTA2 and GAPDH were all designed and synthesized by GenePharma (Shanghai, China).

### Trans-epithelial electrical resistance (TEER) analysis

TEER is one of the commonly used methods to evaluate cell monolayer integrity (Akbari et al. 2017). Caco-2 cells were cultured in a Transwell chamber and then washed with HBSS containing 5 mM HEPES. After that, HBSS/HEPES were added into the basolateral and apical compartment at 1 mL and 0.2 mL volumes and then incubated at 37 °C and 5% CO_2_ for nearly 30 min. The TEER was then transferred to a hot plate at 37 °C and was tested by an epithelial voltmeter with chopstick electrodes (Millicellers-2, Billerica, MA, USA). An insert without cells was used as a blank and its mean resistance was subtracted from all samples, and the TEER value was generally expressed as ohm cm^2^.

### Paracellular permeability analysis

Fluorescein isothiocyanate-dextran (FD) and lucifer yellow (LY) were conducted to assess paracellular permeability. Specifically, the *Staphylococcus_xylosus* and *Staphylococcus_lentus* cultures were co-cultured in a Transwell chamber with the monolayer of Caco-2 cells. The membrane-impermeable tracers LY and FITC conjugated dextran dissolved in PBS were added to the apical compartment and incubated for nearly 4 h, and the medium was added to the basolateral chamber. After the incubation for 1 h at 37 °C, a total of 100 μL solution was collected from the basolateral sides. The transport buffer volume in the apical was 300 μL, and that in the basal sides was 800 μL. A Synergy HTX multimode plate reader was carried out to test the fluorescent signals of FD (the excitation and emission wavelengths were 485 and 530 nm) or LY (the excitation and emission wavelengths were 428 and 540 nm).

### Western blot

The total proteins were extracted from Caco-2 cells using RIPA lysis buffer (Solarbio), and the concentrations of proteins were quantified by a BCA protein assay kit (Abcam, Cambridge, UK). After that, the proteins with different molecular weights were separated by sodium dodecyl sulfate–polyacrylamide gel electrophoresis and then transferred into polyvinylidene fluoride (PVDF) membranes (Thermo Fisher Scientific). The above membranes were then blocked with 5% skimmed milk at room temperature for approximately 1 h, and incubated with the primary antibodies: anti-Claudin-1 (ab211737, 1:2000, Abcam), anti-Claudin-3 (ab214487, 1:1000, Abcam), anti-Occludin (ab216327, 1:1000, Abcam), anti-JAM-1 (ab269948, 1:1000, Abcam) and anti-β-actin (ab8226, 1 µg/mL, Abcam) at 4 °C overnight. The following day, the membranes were incubated with the secondary antibodies for approximately 1 h at room temperature. The protein bands were visualized and quantified using the Enhanced Chemiluminescence Assay Kit (Abcam) and ImageJ software (National Institutes of Health, Bethesda, MD, USA).

### Statistical analysis

All the data were represented as the mean ± SD of three independent assays. The differences between the two groups were evaluated by Student’s *t*-test, and the differences among more than two groups were assessed using one-way analysis of variance (ANOVA). A *P* value of less than 0.05 was considered statistically significant.

## Results

### Resveratrol treatment alleviates CCl_4_-induced liver fibrosis

**Fig. 1:**
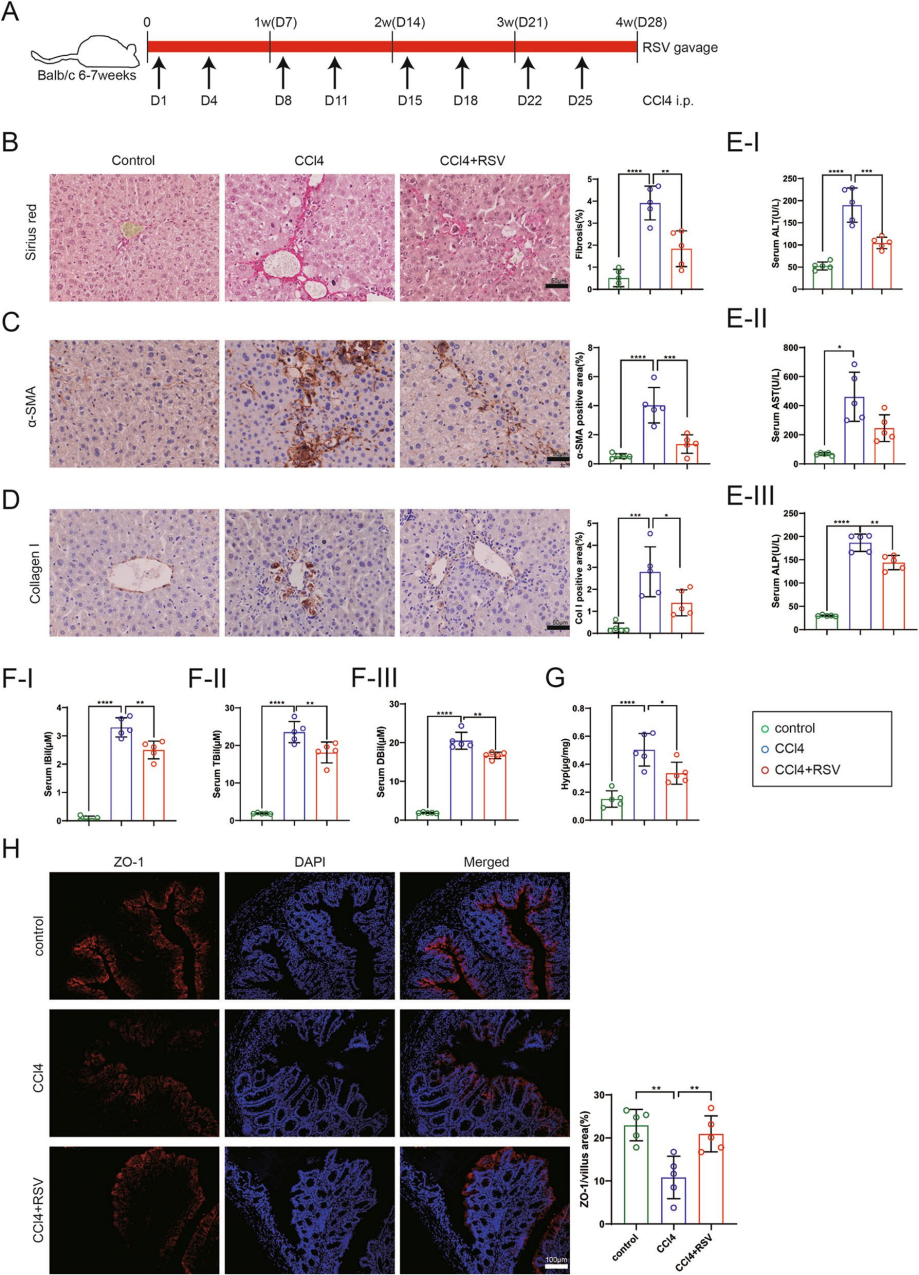
Effect of resveratrol on liver fibrosis induced by CCl_4_. Balb/c male mice (6–7 weeks old) were intraperitoneally injected with olive oil or CCl_4_ (0.5 µL/g) to induce liver fibrosis for 4 weeks twice a week, and the mice were treated with 30 mg/kg resveratrol daily by gavage. **A** A schematic timeline of model mice. **B** Sirius red staining was applied to assess the degree of liver fibrosis in mice (N = 5, scale bar = 50 µm), one-way ANOVA with Tukey’s post-hoc. **C**, **D** The expressions of α-SMA and Collagen I (common markers for liver fibrosis) in mouse liver tissues were measured using immunohistochemical analysis (N = 5), one-way ANOVA with Tukey’s post-hoc. **E**, **F** The levels of (**E**-I) alanine aminotransferase (ALT), (**E**-II) aspartate aminotransferase (AST), (**E**-III) alkaline phosphatase (ALP), (**F**-I) indirect bilirubin (IBil), (**F**-II) total bilirubin (TBil) and (**F**-III) direct bilirubin (DBil) in mouse blood samples were determined using a standard clinical automated analyser, one-way ANOVA with Tukey’s post-hoc or Brown-Forsythe and weltch ANOVA with Games-Howell’s post-hoc. N = 5. **G** Analysis of the hydroxyproline (Hyp) level using a commercial kit, one-way ANOVA with Tukey’s post-hoc. N = 5. **H** The expression of ZO-1 (intercellular adhesion protein) in mouse colon tissues was measured by immunofluorescence staining (scale bar = 100 µm), one-way ANOVA with Tukey’s post-hoc. N = 5. *P < 0.05, **P < 0.01, ***P < 0.001, ****P < 0.0001 vs. Control or CCl_4_ group. Col I: Collagen I. Data are presented of three independent assays

To evaluate the role of resveratrol in liver fibrosis, we constructed a mouse model of liver fibrosis induced by CCl_4_. A schematic timeline of model mice was displayed in Fig. 1A. As exhibited in Fig. 1B and Additional file 4: Fig. S4, the Sirius red staining results expounded that the collagen deposition was increased in the CCl_4_-induced mouse liver tissues, and was reduced after resveratrol treatment, prompting that resveratrol reduced the degree of liver fibrosis. α-SMA and Collagen I are the common markers for liver fibrosis. The immunohistochemical analysis demonstrated that α-SMA and Collagen I were highly expressed in the CCl_4_-induced mouse liver tissues, and were decreased after resveratrol treatment (Fig. 1C, D and Additional file 4: Fig. S4).

ALT, AST, ALP, IBiL, TBiL and DBiL are common parameters for liver function detection. The levels of ALT, AST, ALP, IBiL, TBiL and DBiL were elevated in the CCl_4_-induced mouse blood samples, while the resveratrol treatment partially reversed this trend (Fig. 1E, F). Hydroxyproline (Hyp) is a marker for liver fibrosis (Ambade et al. 2019). As displayed in Fig. 1G, the Hyp was elevated in the CCl_4_ group in comparison with the control group, while this effect was partially reversed by the resveratrol treatment. Meanwhile, the expression of ZO-1 (intercellular adhesion protein) in mouse colon tissues was assessed and the results demonstrated that resveratrol repressed the loss of ZO-1 induced by CCl_4_ (Fig. 1H). In summary, the above results corroborated that administration of resveratrol ameliorated CCl_4_-induced liver injury in mice and accelerated the repair of the intestinal mucosal barrier.

### Resveratrol alters gut microbiota in the progression of liver fibrosis in mice

**Fig. 2:**
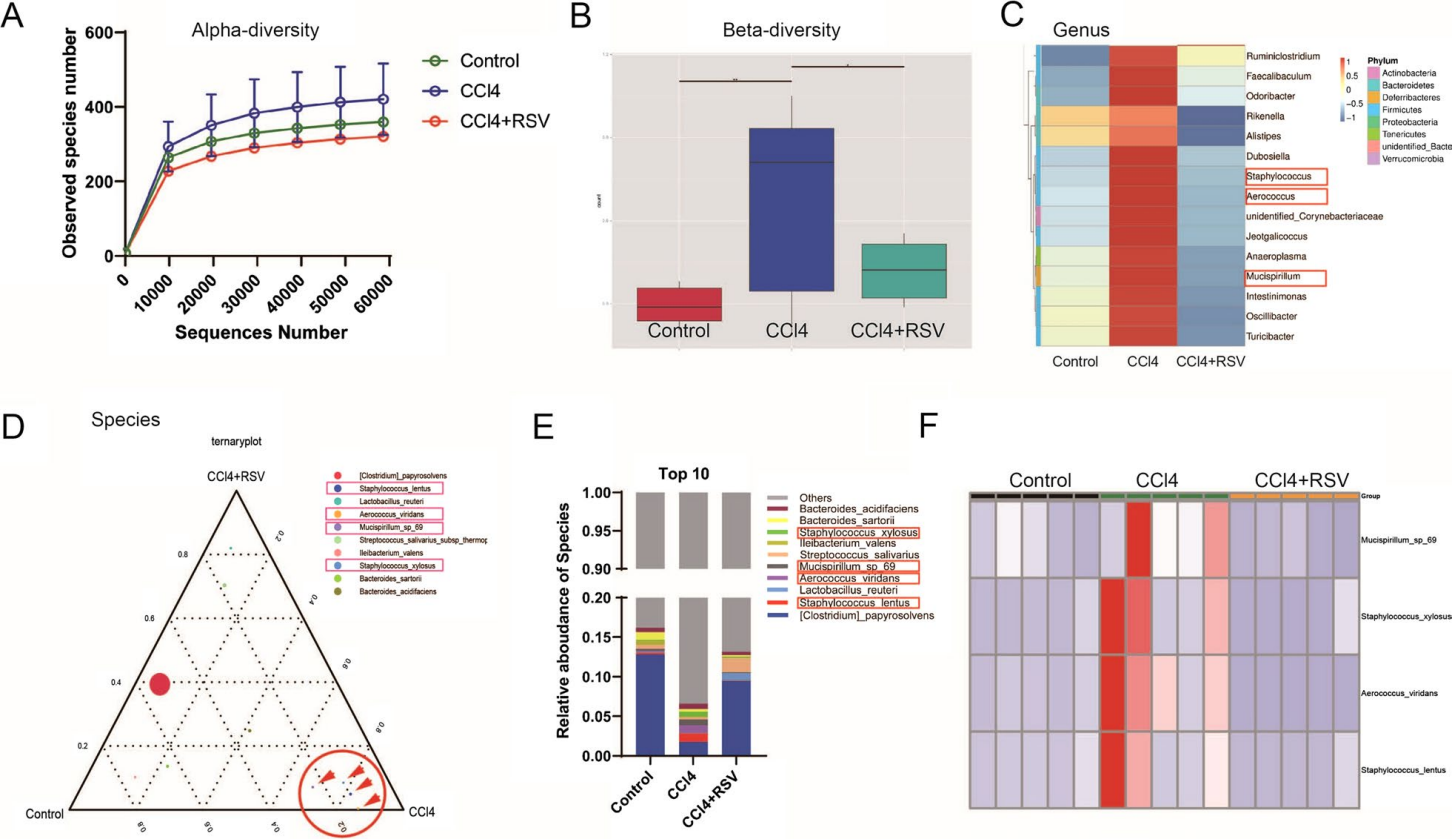
Influence of resveratrol on gut microbiota in the progression of liver fibrosis in mice. Balb/c male mice (6–7 weeks old) were intraperitoneally injected with olive oil or CCl_4_ (0.5 µL/g) to induce liver fibrosis for 4 weeks twice a week, and the mice were treated with 30 mg/kg resveratrol daily by gavage. Feces were collected for 16S rRNA sequencing in the fourth week. **A**, **B** The diversity and composition of microbial communities were evaluated using alpha-diversity and beta-diversity analysis, one-way ANOVA with Tukey’s post-hoc. **C** Genus analysis. **D**, **E** The dominant bacterial communities were assessed using Ternaryplot analysis and Top Ten abundance on species. **F** Analysis of the relative abundance of species, Z-score. *P < 0.05, **P < 0.01 vs. Control or CCl_4_. Data are presented of three independent assays

Previous research corroborates that resveratrol alleviates nonalcoholic fatty liver disease by decreasing the abundance of harmful bacteria in the gut microbiota (Wang 2020). As exhibited in Fig. 2A, B, alpha-diversity and beta-diversity were significantly elevated after the CCl_4_ treatment, indicative an increase of the diversity and composition of microbial community, while the resveratrol treatment partially reversed these trends. Thus, we hypothesized that resveratrol obtained its therapeutic function by reducing some harmful bacteria.

Subsequently, the changes of gut microbiota in mice after CCl_4_ induction and resveratrol intervention were assessed. Compared with CCl_4_ treatment group, 15 bacterial genus with repressed abundance were obtained in the resveratrol and CCl_4_ treatment group, which were exhibited in a heat map (Fig. 2C). Meanwhile, according to the Ternaryplot analysis and Top Ten abundance on species, four dominant bacterial communities (Mucispirillum_sp_69, *Staphylococcus_xylosus*, *Staphylococcus_lentus* and *Aerococcus_viridans*) were pronounced after CCl_4_ induction, and they were inhibited after the treatment of resveratrol (Fig. 2D, E). Among the above four bacterial communities, remarkable decreases of abundance on *Staphylococcus_xylosus*, *Staphylococcus_lentus* and *Aerococcus_viridans* were observed in resveratrol and CCl_4_ treatment group (Fig. 2F). Thus, these bacterial communities were taken as the main research objects to further investigate their effects on liver fibrosis progression. Moreover, the preliminary study on the mechanism of resveratrol restraining *Staphylococcus* indicated that resveratrol restrained the proliferation of *Staphylococcus* by repressing the formation of *Staphylococcus* biofilms (Additional file 1: Fig. S1).

### *Staphylococcus_xylosus* and *Staphylococcus_lentus* facilitate the development of liver fibrosis in mice

**Fig. 3:**
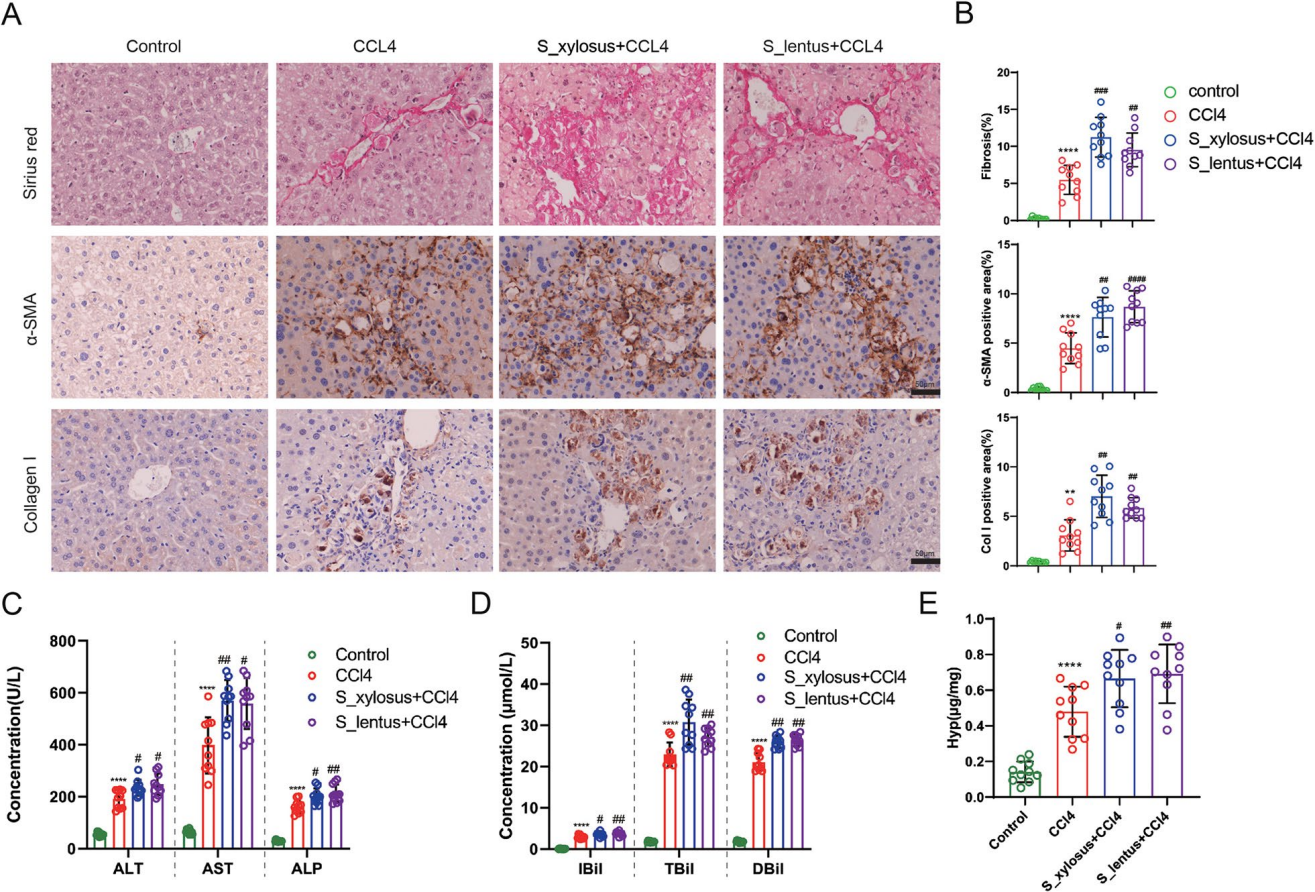
Regulation of *Staphylococcus_xylosus* and *Staphylococcus_lentus* on the process of liver fibrosis in mice. The pure cultures (10^9^ CFU) of the three kinds of gut microbiota (*Staphylococcus_lentus*, *Aerococcus_viridans* and *Staphylococcus_xylosus*) were given to mice by gavage to make them become the dominant microflora in the intestine, and then the liver fibrosis model of mice was induced by the intraperitoneal injection of CCl_4_ (0.5 µL/g). **A**, **B** Sirius red staining and immunohistochemical assays were conducted to assess the degree of liver fibrosis in mice (N = 10, scale bar = 50 µm), Brown-Forsythe and Welch ANOVA with Games-Howell’s post-hoc. **C** Different detection kits were applied to measure the concentrations of ALT, AST and ALP in mouse blood samples, Brown-Forsythe and Welch ANOVA with Games-Howell’s post-hoc. N = 10. **D** The concentrations of IBiL, TBiL and DBiL in mouse blood samples were assessed, one-way ANOVA with Tukey’s post-hoc for IBil, Brown-Forsythe and Welch ANOVA with Games-Howell’s post-hoc for TBil and DBil. N = 10. **E** Comparison of the hydroxyproline (Hyp) level using a commercial kit. N = 10. **P < 0.01, ****P < 0.0001 vs. Control. ^#^*P* < 0.05, ^##^*P* < 0.01, ^###^*P* < 0.001, ^####^*P* < 0.0001 vs. CCl_4_. *S_xylosus*: *Staphylococcus_xylosus*, *S_lentus*: *Staphylococcus_lentus*, *A_viridans*: *Aerococcus_viridans*. Data are presented of three independent assays

To further investigate the functions of *Staphylococcus_xylosus* and *Staphylococcus_lentus* and *Aerococcus_viridans* in CCl_4_-induced liver fibrosis, the pure cultures of them were administered to mice to make them to be the dominant microflora in the intestine, and then the mouse liver fibrosis model was induced by CCl_4_. The results of Sirius red staining confirmed that the degree of liver fibrosis was more serious in the CCl_4_ group, and the gavage of *Staphylococcus_xylosus* and *Staphylococcus_lentus* further aggravated the liver fibrosis in mice (Fig. 3A, B and Additional file 4: Fig. S4), while the gavage of *Aerococcus_viridans* had no significant effect on liver fibrosis in mice compared with CCl_4_ induction group (data was not shown). Thus, the following assays were performed on *Staphylococcus_xylosus* and *Staphylococcus_lentus* treatment group. The α-SMA and Collagen I were elevated in the mouse liver tissues by IHC staining in the CCl_4_ group, and this trend was further enhanced after the gavage of the *Staphylococcus_xylosus* and *Staphylococcus_lentus* pure cultures (Fig. 3A, B). The gavage of *Staphylococcus_xylosus* and *Staphylococcus_lentus* further increased the concentrations of ALT, AST, ALP, IBiL, TBiL and DBiL in the mouse blood samples (Fig. 3C, D). The Hyp was further elevated after the gavage of *Staphylococcus_xylosus* and *Staphylococcus_lentus* (Fig. 3E). Besides, the indexes of *Aerococcus_viridans* were not significantly different from those in the CCl_4_ group (data were not shown), so the follow-up studies were mainly conducted on the *Staphylococcus_xylosus* and *Staphylococcus_lentus*. Conclusively, *Staphylococcus_xylosus* and *Staphylococcus_lentus* aggravated liver fibrosis in mice.

### *Staphylococcus_xylosus* and *Staphylococcus_lentus* promote the occurrence of bacterial translocation

**Fig. 4:**
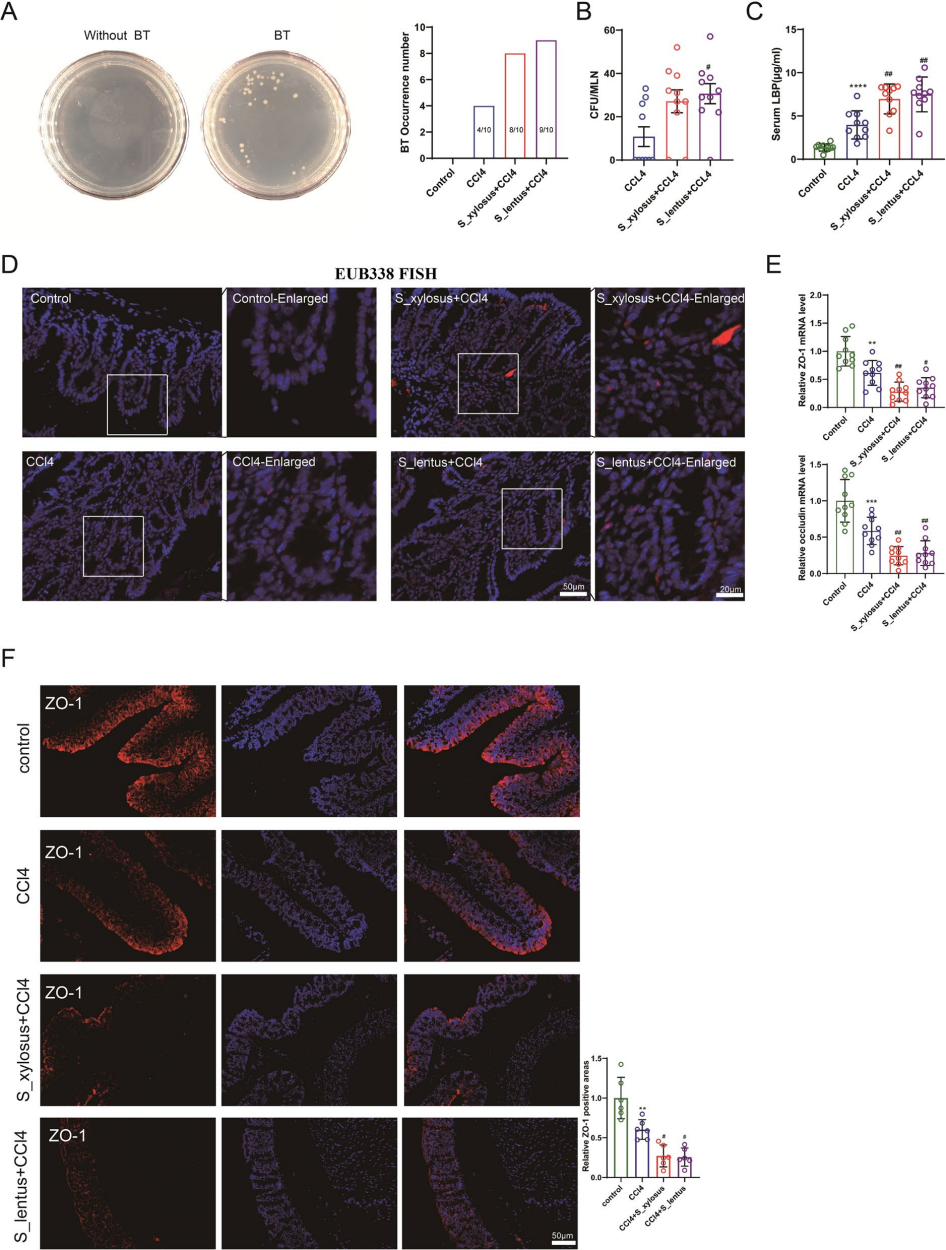
Influence of *Staphylococcus_xylosus* and *Staphylococcus_lentus* in the occurrence of BT. The pure cultures (10^9^ CFU) of the two kinds of gut microbiota (*Staphylococcus_lentus* and *Staphylococcus_xylosus*) were given to mice by gavage to make them become the dominant microflora in the intestine, and then the liver fibrosis model of mice was induced by intraperitoneal injection of CCl_4_ (0.5 µL/g). **A** After bacterial colony culture on mesenteric lymph nodes (MLNs), the occurrence of the colonies was observed. **B** Statistical analysis on the number of bacterial colonies, one-way ANOVA with Tukey’s post-hoc. N = 10. **C** Analysis of the concentration of LBP in the mouse blood samples by a commercial kit. N = 10. **D** The occurrence of BT was assessed using EUB338 FISH detection. N = 10. **E** The mRNA levels of zonula occludens-1 (ZO-1) and occludin were measured by qRT-PCR. N = 10. **F** The relative ZO-1 positive areas were measured using immunofluorescence (scale bar = 50 µm), one-way ANOVA with Tukey’s post-hoc. N = 10. **P < 0.01, ***P < 0.001, ****P < 0.0001 vs. Control. ^#^*P* < 0.05, ^##^*P* < 0.01 vs. CCl_4_. Data are presented of three independent assays

BT is a process by which intestinal bacteria or the products cross the intestinal barrier and enter MLNs or other extraintestinal tissues (Shi et al. 2017). Combined with the finding that the numerical expressions of ALP, TBil and DBil in blood test indexes were similar to the above references, we speculated that *Staphylococcus_xylosus* and *Staphylococcus_lentus* might accelerate the occurrence of BT. The occurrence of BT could be observed by the bacterial colony culture of MLNs. As displayed in Fig. 4A, B, BT occurred when a bacterial colony appeared after bacterial colony culture on MLNs.

LBP is one of the molecules that indicate the occurrence of BT. Thus, we evaluated the LBP level in mouse blood samples and the results corroborated that the LBP level was further elevated after the gavage of *Staphylococcus_xylosus* and *Staphylococcus_lentus* (Fig. 4C). EUB338 is a universal probe for bacteria. When BT occurs, the bacteria cross the intestinal mucosa and enter the lamina propria (Huang et al. 2020). Thus, if there is red fluorescence in the lamina propria, it is considered BT positive. As exhibited in Fig. 4D, the gavage of *Staphylococcus_xylosus* and *Staphylococcus_lentus* both accelerated the BT occurrence.

ZO-1 and occludin characterize the stability of the intestinal mucosal barrier, which is one of the most important causes of intestinal BT (Wang, et al. 2019; Joly Condette 2014). As shown in Fig. 4E, the ZO-1 and occludin were further decreased after the gavage of *Staphylococcus_xylosus* and *Staphylococcus_lentus*. The immunofluorescence analysis of ZO-1 expression showed the same trend (Fig. 4F). Meanwhile, the *Staphylococcus_xylosus* and *Staphylococcus_lentus* were gavage to normal mice. Sirius Red training and Hyp detection showed no obvious changes (Additional file 2: Fig. S2A, B). While the mRNA level of ACTA2 (encoded α-SMA) was significantly increased after the gavage of *Staphylococcus_xylosus* and *Staphylococcus_lentus* (Additional file 2: Fig. S2C), and ZO-1 was slightly decreased (Additional file 2: Fig. S2D). In addition, the pro-inflammatory cytokine TNF-α and IL-6 in the liver was measured after treatment of *Staphylococcus_xylosus* or *Staphylococcus_lentus* in CCl_4_-induced mice, and the result showed that *Staphylococcus_xylosus* or *Staphylococcus_lentus* treatment significantly upregulated the protein levels of TNF-α and IL-6 (Additional file 3: Fig. S3). Therefore, we believed that the gavage of *Staphylococcus_xylosus* or *Staphylococcus_lentus* alone had a limited impact on the liver injury of normal mice, and its effect on the deterioration of the disease was more remarkable in the case of CCl_4_-induced lesions.

### The cultures of *Staphylococcus_xylosus* and *Staphylococcus_lentus* change the permeability of intestine

**Fig. 5:**
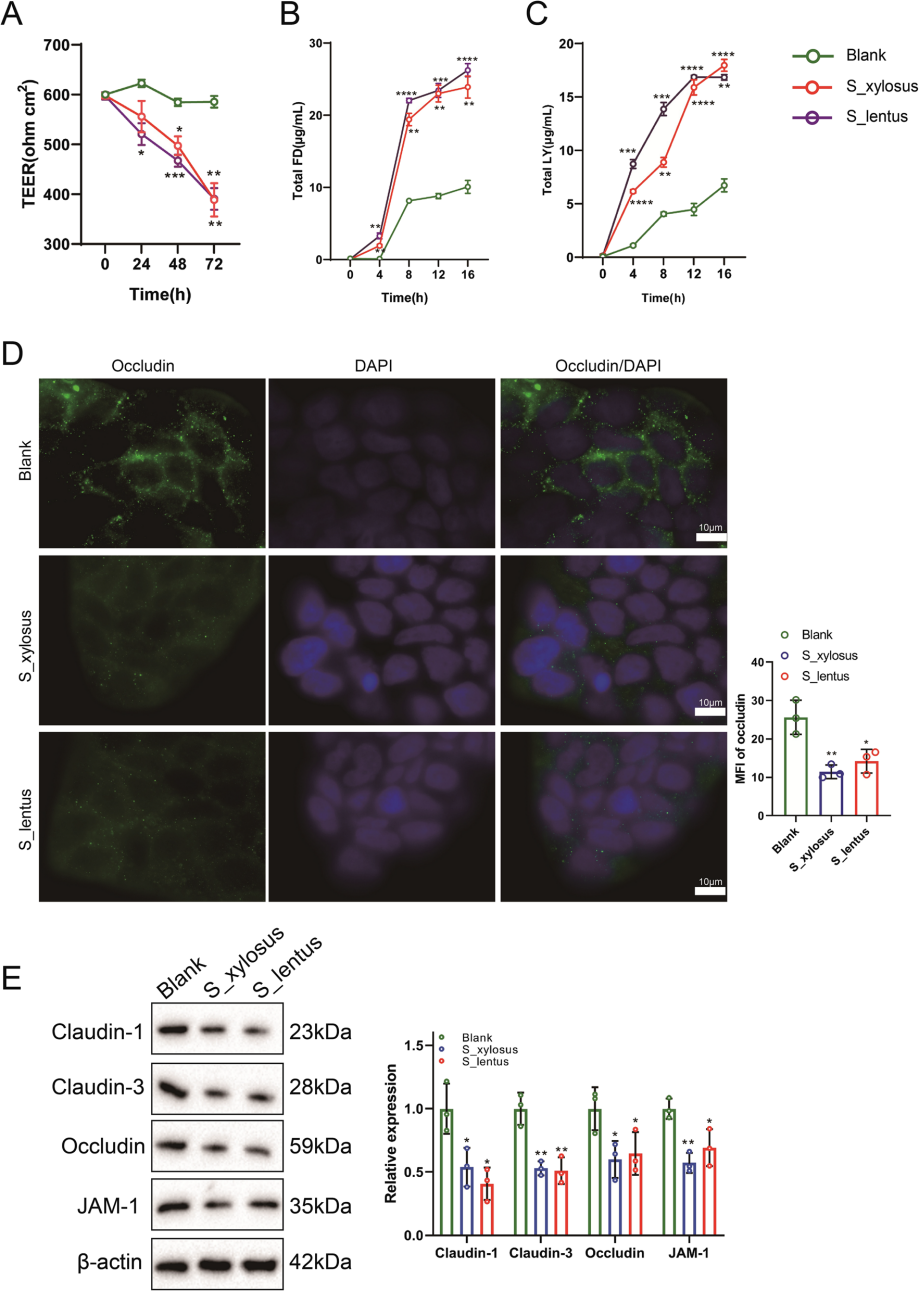
Change of the cultures of *Staphylococcus_xylosus* and *Staphylococcus_lentus* on the permeability of intestine. The *Staphylococcus_xylosus* or *Staphylococcus_lentus* was cultured with Caco-2 cells. **A** Trans-epithelial electrical resistance (TEER) analysis was applied to assess cell permeability, two-way ANOVA with Tukey’s post-hoc. **B** The fluorescein isothiocyanate-dextran (FD) analysis was applied to measure the paracellular permeability, two-way ANOVA with Tukey’s post-hoc. **C** Lucifer yellow (LY) was conducted to assess the cell permeability, two-way ANOVA with Tukey’s post-hoc. **D** Immunofluorescence assay was applied to measure the mean fluorescence intensity (MFI) of occludin (scale bar = 10 µm), one-way ANOVA with Tukey’s post-hoc. **E** Western blot and quantitative real-time PCR (qRT-PCR) were applied to quantify the protein and mRNA levels of Claudin-1, Claudin-3, Occludin and JAM-1, one-way ANOVA with Tukey’s post-hoc. *P < 0.05, **P < 0.01, ***P < 0.001, ****P < 0.0001 vs. Blank. Data are presented of three independent assays

The cultures of *Staphylococcus_xylosus* and *Staphylococcus_lentus* were cultured together with Caco-2 cells to assess the changes in cell permeability. The cell permeability was analyzed and the data revealed that the TEER value was lessened after adding the cultures of *Staphylococcus_xylosus* and *Staphylococcus_lentus* (Fig. 5A). The fluorescein isothiocyanate-dextran (FD) analysis expounded that the content of total FD was increased after adding the cultures of *Staphylococcus_xylosus* and *Staphylococcus_lentus* (Fig. 5B). Similarly, the analysis of lucifer yellow (LY) indicated that the content of LY was elevated after adding the cultures of *Staphylococcus_xylosus* and *Staphylococcus_lentus* (Fig. 5C), hinting that the addition of the cultures of *Staphylococcus_xylosus* and *Staphylococcus_lentus* increased the permeability of the intestine. Immunofluorescence results expounded that the mean fluorescence intensity (MFI) of occludin (a tight junction protein) was lessened after adding the cultures of *Staphylococcus_xylosus* and *Staphylococcus_lentus* (Fig. 5D). Claudin-1, Claudin-3, Occludin, and JAM-1 are tight junction proteins, which are interrelated to the permeability of the intestine (Xiao et al. 2016). As exhibited in Fig. 5E, the protein and mRNA levels of Claudin-1, Claudin-3, Occludin, and JAM-1 were decreased after adding the cultures of *Staphylococcus_xylosus* and *Staphylococcus_lentus*. Overall, the cultures of *Staphylococcus_xylosus* and *Staphylococcus_lentus* enhanced the permeability of the intestine.

### Excessive *Staphylococcus_xylosus* and *Staphylococcus_lentus* counteract the improvement of resveratrol on liver fibrosis progression

**Fig. 6:**
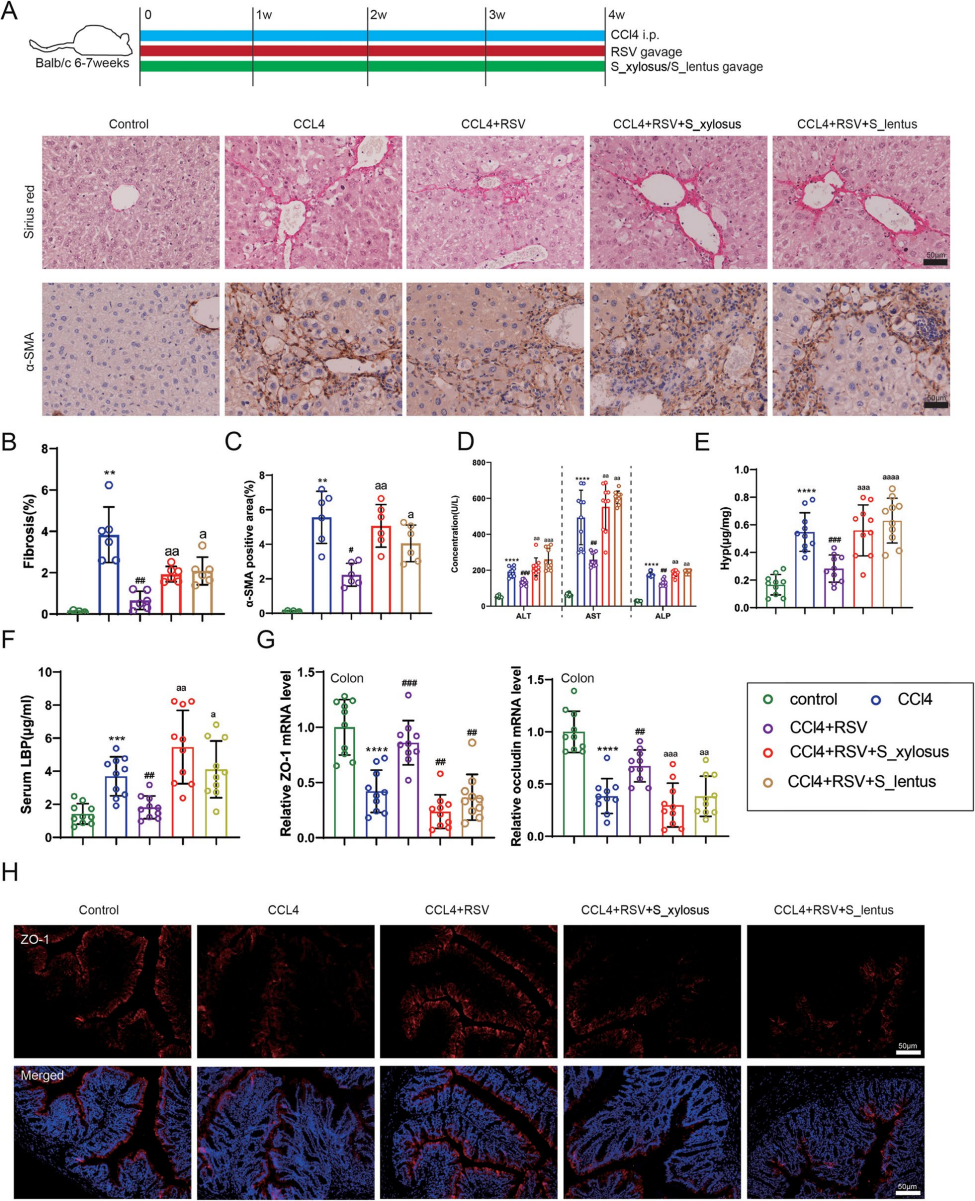
Influence of *Staphylococcus_xylosus* and *Staphylococcus_lentus* on the improvement of resveratrol on liver fibrosis. The pure cultures of the *Staphylococcus_lentus* and *Staphylococcus_xylosus* (10^9^ CFU) were given to mice by gavage, and then the liver fibrosis model of mice was induced by the intraperitoneal injection of CCl_4_ (0.5 µL/g), and the mice were treated with resveratrol (30 mg/kg) daily by gavage. **A** The protocol for in vivo assays, and representative images of Sirius red staining (scale bar = 50 µm) and Immunohistochemical staining (scale bar = 50 µm) of α-SMA on mouse liver tissues. N = 6. **B** Quantitative results for the Sirius red staining. Brown-Forsythe and Welch ANOVA with Games-Howell’s post-hoc, Mean ± SD, N = 6. **C** Quantitative results for the Immunohistochemical staining of α-SMA. Brown-Forsythe and Welch ANOVA with Games-Howell’s post-hoc, Mean ± SD, N = 6. **D** The concentrations of ALT, AST and ALP in the mouse blood samples were measured using different detection kits, ALT, AST: Brown-Forsythe and Welch ANOVA with Games-Howell’s post-hoc; ALP: one-way ANOVA with Tukey's post-hoc, Mean ± SD, N = 10. **E** Comparison of the Hyp level by a commercial kit, one-way ANOVA with Tukey’s post-hoc, Mean ± SD, N = 10. **F** Analysis of the concentration of LBP in the mouse blood samples using a commercial kit, Brown-Forsythe and Welch ANOVA with Games-Howell’s post-hoc, Mean ± SD, N = 10. **G** The mRNA levels of ZO-1 and occludin were assessed by qRT-PCR. N = 10. **H** The relative ZO-1 positive areas were measured using immunofluorescence (scale bar = 50 µm). N = 6. **P < 0.01, ***P < 0.001, ****P < 0.0001 vs. Control. ^#^*P* < 0.05, ^##^*P* < 0.01, ^###^*P* < 0.001 vs. CCl_4_. ^a^*P* < 0.05, ^aa^*P* < 0.01, ^aaa^*P* < 0.001, ^aaaa^*P* < 0.0001 vs. CCl_4_ + resveratrol. Data are presented of three independent assays

To investigate the regulation of *Staphylococcus_xylosus* and *Staphylococcus_lentus* on resveratrol in alleviating liver fibrosis, the pure cultures of the *Staphylococcus_lentus* and *Staphylococcus_xylosus* were given to mice by gavage, and then the liver fibrosis model of mice was induced by intraperitoneal injection of CCl_4_, and the mice were treated with resveratrol daily by gavage. The protocol for in vivo assays was presented in Fig. 6A and Additional file 4: Fig. S4. From the results of Sirius red staining, we confirmed that the resveratrol treatment alleviated the liver fibrosis in mice, while the gavage of *Staphylococcus_lentus* and *Staphylococcus_xylosus* aggravated the liver fibrosis in mice (Fig. 6B). Meanwhile, immunohistochemical analysis revealed that the resveratrol treatment decreased the α-SMA expression, while this decrease was partially reversed after the gavage of *Staphylococcus_lentus* and *Staphylococcus_xylosus* (Fig. 6B), and the quantitative results for the Sirius red staining and immunohistochemistry were exhibited in Fig. 6B, C. Furthermore, the resveratrol treatment reduced the concentrations of ALT, AST and ALP, while the gavage of *Staphylococcus_lentus* and *Staphylococcus_xylosus* reversed this reduction (Fig. 6D). These results suggested that resveratrol reduced the blood parameters of CCl_4_-induced mice and the gavage of *Staphylococcus_lentus* and *Staphylococcus_xylosus* aggravated the mouse liver injury. From the sequencing results, resveratrol repressed the proliferation of these two bacteria and the effects of resveratrol could be offset by increasing the levels of these two bacteria by gavage.

Besides, to investigate whether the intestinal mucosal barrier was changed, the Hyp level was assessed and the results indicated that the resveratrol treatment reduced the Hyp level, while this reduction was partially reversed after the gavage of *Staphylococcus_lentus* and *Staphylococcus_xylosus* (Fig. 6E). The analysis of LBP exhibited a similar trend (Fig. 6F). Meanwhile, the ZO-1 and occludin were increased after the resveratrol treatment and were decreased after the gavage of *Staphylococcus_xylosus* and *Staphylococcus_lentus* (Fig. 6G) and the results of immunofluorescence analysis of ZO-1 showed the same trend (Fig. 6H). Conclusively, the *Staphylococcus_xylosus* and *Staphylococcus_lentus* reversed the improvement of resveratrol on liver fibrosis.

## Discussion

The current study aimed to clarify the mechanism by which resveratrol regulated the pathological progress in liver fibrosis. Here, we discovered that resveratrol restrained the liver fibrosis induced by CCl_4_, which was consistent to the descriptions in previous literatures (Hessin et al. 2017; Zhang et al. 2016). Combined with the facilitating effect of gut microbiota on liver fibrosis (Zhou et al. 2019), we next explored the potential mechanism and corroborated that resveratrol altered the gut microbiota in liver fibrosis. The further data revealed that *Staphylococcus_xylosus* and *Staphylococcus_lentus* induced BT and their cultures enhanced the permeability of intestine. More important, the in vivo assay confirmed that the excessive *Staphylococcus_xylosus* and *Staphylococcus_lentus* canceled the amelioration of resveratrol on liver fibrosis.

It is well-known that resveratrol exerts the effect of anti-liver fibrosis (Zhao et al. 2017; Ahmad and Ahmad 2014). As expected, our experimental data showed that administration of resveratrol relieved CCl_4_-induced liver fibrosis. Thus, this study investigated the mechanisms by which resveratrol functioned its role in liver fibrosis. Gut microbiota is a novel kind of virtual metabolic organ, which maintains homeostasis in the host; when the homeostasis is disrupted, the gut microbiota and its metabolites migrate to the liver, which in turn induces a range of liver diseases, such as liver fibrosis (Ohtani and Kawada 2019; Konturek, et al. 2018). Accumulating evidences clarify that resveratrol regulates various human diseases through affecting the composition of gut microbiota. For instance, Wang et al. have claimed that resveratrol improves nonalcoholic fatty liver disease (NAFLD) by reducing the abundance of harmful bacteria in the gut microbiota (Wang 2020). Chen et al. have expounded that resveratrol improves NAFLD by altering the composition of gut microbiota and maintaining the integrity of the intestinal barrier (Chen et al. 2020). In order to identify the effect of resveratrol on gut microbiota, the feces of mice were collected for the 16S rRNA sequencing. Our results indicated that resveratrol administration reduced the abundance of gut microbiota including Mucispirillum_sp_69, *Staphylococcus_xylosus*, *Staphylococcus_lentus*, and *Aerococcus_viridans* in CCl_4_-induced model mice, which were consistent to the previous studies. Among the four dominant bacterial communities, the in vivo data suggested that *Staphylococcus_xylosus* and *Staphylococcus_lentus* treatment aggravated the liver fibrosis. Interestingly, several studies demonstrated that *Staphylococcus* was recognized as an important pathogen in patients with chronic liver diseases and was closely related to the occurrence of cystic fibrosis (Obeidat 2021; Kang et al. 2010), which were similar to us and may partly support our data. In fact, the potential mechanisms of resveratrol affecting liver fibrosis are complex. Up to now, studies have shown that there are many factors affecting liver fibrosis under resveratrol treatment, including the key signaling pathways, such as the hippo pathway and PI3K/AKT pathway, and the anti-oxidative and anti-inflammatory effects(Yu 2019; Chen et al. 2020; Hessin et al. 2017; Vairappan et al. 2019). The molecular mechanism we provided in our work enriched the resveratrol therapeutic discovery on liver fibrosis.

Increasing evidences suggest that gut microbiota influence liver diseases mainly through the BT pathway. For instance, Chen et al. confirmed that the traditional Chinese medicine artesunate restrains BT and reduces inflammation by regulating gut microbiota, thereby reducing CCl_4_-induced liver and intestinal injury(Chen et al. 2016). Hackstein et al. have indicated that gut microbiota induces high level of IFN through BT, which in turn destroys bacterial immunity and leads to severe liver fibrosis (Hackstein et al. 2017). In this work, the data authenticated that *Staphylococcus_xylosus* and *Staphylococcus_lentus* facilitated the occurrence of BT, which was consistent with the above findings. Besides, previous studies have clarified that the increased intestinal permeability facilitates the migration of gut microbiota from the gut to the liver, hinting that intestinal permeability is one of the key factors affecting BT(Ma et al. 2018). Similar to this evidence, our study discovered that the cultures of *Staphylococcus_xylosus* and *Staphylococcus_lentus* promoted the permeability of intestine. Meanwhile, the in vivo assay further revealed that the excessive *Staphylococcus_xylosus* and *Staphylococcus_lentus* reversed the improvement of resveratrol on liver fibrosis. In general, BT is a factor that triggers various mechanisms in the liver damage that proceeds fibrosis, including activation of Kupffer cells, the deposition of lipids, and chronic inflammation, et al. This study confirms that BT occurs in the presence of *Staphylococcus_xylosus* and *Staphylococcus_lentus* during liver fibrosis, whereas the exact mechanism that BT facilitates fibrosis is not fully known and needs to be further explored.

Overall, our results clarified that resveratrol ameliorated liver fibrosis by reducing the gut microbiota *Staphylococcus_xylosus* and *Staphylococcus_lentus*, which might be a novel therapeutic direction for liver fibrosis. Based on this finding, we further confirmed that the *Staphylococcus_xylosus* and *Staphylococcus_lentus* accelerated BT, enhanced the permeability of intestine, and weakened the ameliorative effect of resveratrol on liver fibrosis (Additional file 5: Fig. S5). Our study may provide novel insights for relieving liver fibrosis. At present, we are in the preliminary stage in exploring the mechanism of resveratrol repressing *Staphylococcus*, and we will investigate it profoundly to improve the integrity of this work in the next direction.

## Supplementary Information

**Additional file 1: Figure S1.** Resveratrol restrains the formation of *Staphylococcus_xylosus* and *Staphylococcus_lentus* biofilms. (A-B) *Staphylococcus_xylosus* and *Staphylococcus_lentus* were treated with resveratrol with the minimum inhibitory concentration (MIC, 260 μg/mL), 1/2 MIC, 1/4 MIC and 1/8 MIC for 0, 1, 2, 3, 4, 5, 6, 7, 8, 9, 10 h. Growth curves were applied to analyze the restraint of resveratrol on *Staphylococcus_xylosus* and *Staphylococcus_lentus* with different concentrations. (C-D) *Staphylococcus_xylosus* and *Staphylococcus_lentus* and their biofilms were treated with 1U of penicillin (PNC), and then the *Staphylococcus_xylosus* *Staphylococcus_lentus* biofilms were further treated with 1/8 MIC for 0, 1, 2, 3, 4, 5, 6, 7, 8, 9, 10 h. Growth curves were applied to analyze the synergistic effects of resveratrol and PNC on the inhibition of two types of *Staphylococcus* that induced biofilm formation. *P < 0.05 vs. S_xylosus or S_lentus group. **P < 0.01 vs. Blank, S_xylosus or S_lentus group, ^##^*P* < 0.01 vs. S_xylosus + PNC or S_lentus + PNC group, ^aa^*P* < 0.01 vs. S_xylosus (biofilm) + PNC or S_lentus (biofilm) + PNC. Data are presented of three independent experiments and expressed as mean ± SD.

**Additional file 2: Figure S2.** Evaluation of *Staphylococcus_xylosus* and *Staphylococcus_lentus* on liver injury in normal mice. The pure cultures of the *Staphylococcus_lentus* and *Staphylococcus_xylosus* (10^9^ CFU) were given to normal mice by gavage. (A) The protocol for in vivo assays. The degree of liver fibrosis in mice was evaluated by Sirius red staining, one-way ANOVA with Tukey’s post-hoc. N = 5. (B) The Hyp level was detected by an ELISA kit, one-way ANOVA with Tukey's post-hoc, Mean ± SD, N = 6. (C) The mRNA level of ACTA2 (encoded α-SMA) was determined by qRT-PCR, one-way ANOVA with Tukey’s post-hoc. N = 6. (D) The relative ZO-1 positive areas were measured using immunofluorescence (scale bar = 50 µm). N = 5. *P < 0.05, ***P < 0.001 vs. Control. Data are presented of three independent assays.

**Additional file 3: Figure S3.** Effects of *Staphylococcus_xylosus* and *Staphylococcus_lentus* on pro-inflammatory cytokine levels in the liver of CCl_4_-induced mice. Mice received the pure cultures of the *Staphylococcus_lentus* or *Staphylococcus_xylosus* (10^9^ CFU) by gavage. The intraperitoneal injection of CCl_4_ (0.5 µL/g) was performed to induce liver fibrosis model followed by the treatment of resveratrol (30 mg/kg) daily by gavage. The pro-inflammatory cytokine TNF-α and IL-6 in the liver was measured by western blotting. N = 6.

**Additional file 4: Figure S4.** The additional representative images of Sirius red staining and immunohistochemical staining. (F1B-F1D) Sirius red staining was performed to assess the damage of liver fibrosis in mice. N = 5, scale bar = 50 µm. The expressions of α-SMA and Collagen I in mouse liver tissues were measured using immunohistochemical analysis. N = 5, scale bar = 50 µm. (F3A) Sirius red staining and immunohistochemical assays of α-SMA and Collagen I were conducted to assess liver fibrosis in mice. N = 10, scale bar = 50 µm. (F6A) Images of Sirius red staining (scale bar = 50 µm) and Immunohistochemical staining of α-SMA on mouse liver tissues. N = 6. scale bar = 50 µm.

**Additional file 5: Figure S5.** A schematic representation for resveratrol in regulating liver fibrosis. The resveratrol treatment reduced the abundance of *Staphylococcus_xylosus* and *Staphylococcus_lentus*, which were crucial for the development of liver fibrosis. *Staphylococcus_xylosus* and *Staphylococcus_lentus* promoted the occurrence of bacterial translocation via enhancing the permeability of intestine, while resveratrol treatment reversed the effect and eventually ameliorated liver fibrosis.

## Data Availability

All data generated or analyzed during this study are included in this published article.

## Abbreviations

BT: Bacterial translocation
MLNs: Mesenteric lymph nodes
rRNA: Ribosomal RNA
ALT: Alanine aminotransferase
AST: Aspartate aminotransferase
ALP: Alkaline phosphatase
IBil: Indirect bilirubin
TBil: Total bilirubin
DBil: Direct bilirubin
GGT: Glutamyl transpeptidase
ATCC: American type culture collection
TSA: Trypticase soy agar
TSB: Trypticase soy brothx
MIC: Minimum inhibitory concentration
PNC: Penicillin
LBP: Lipopolysaccharide-binding protein
ZO-1: Zonula occludens-1
TEER: Trans-epithelial electrical resistance
FD: Fluorescein isothiocyanate-dextran
LY: Lucifer yellow
SDS-PAGE: Sodium dodecyl sulfate–polyacrylamide gel electrophoresis
PVDF: Polyvinylidene fluoride
qRT-PCR: Quantitative real-time PCR
ANOVA: One-way analysis of variance
MFI: Mean fluorescence intensity
NAFLD: Nonalcoholic fatty liver disease
