# Supplementary figures and images for: Resveratrol ameliorates liver fibrosis induced by nonpathogenic Staphylococcus in BALB/c mice through inhibiting its growth
Source: Mol Med. 2022 May 4;28:52. doi: 10.1186/s10020-022-00463-y (PMC9066969; doi:10.1186/s10020-022-00463-y)

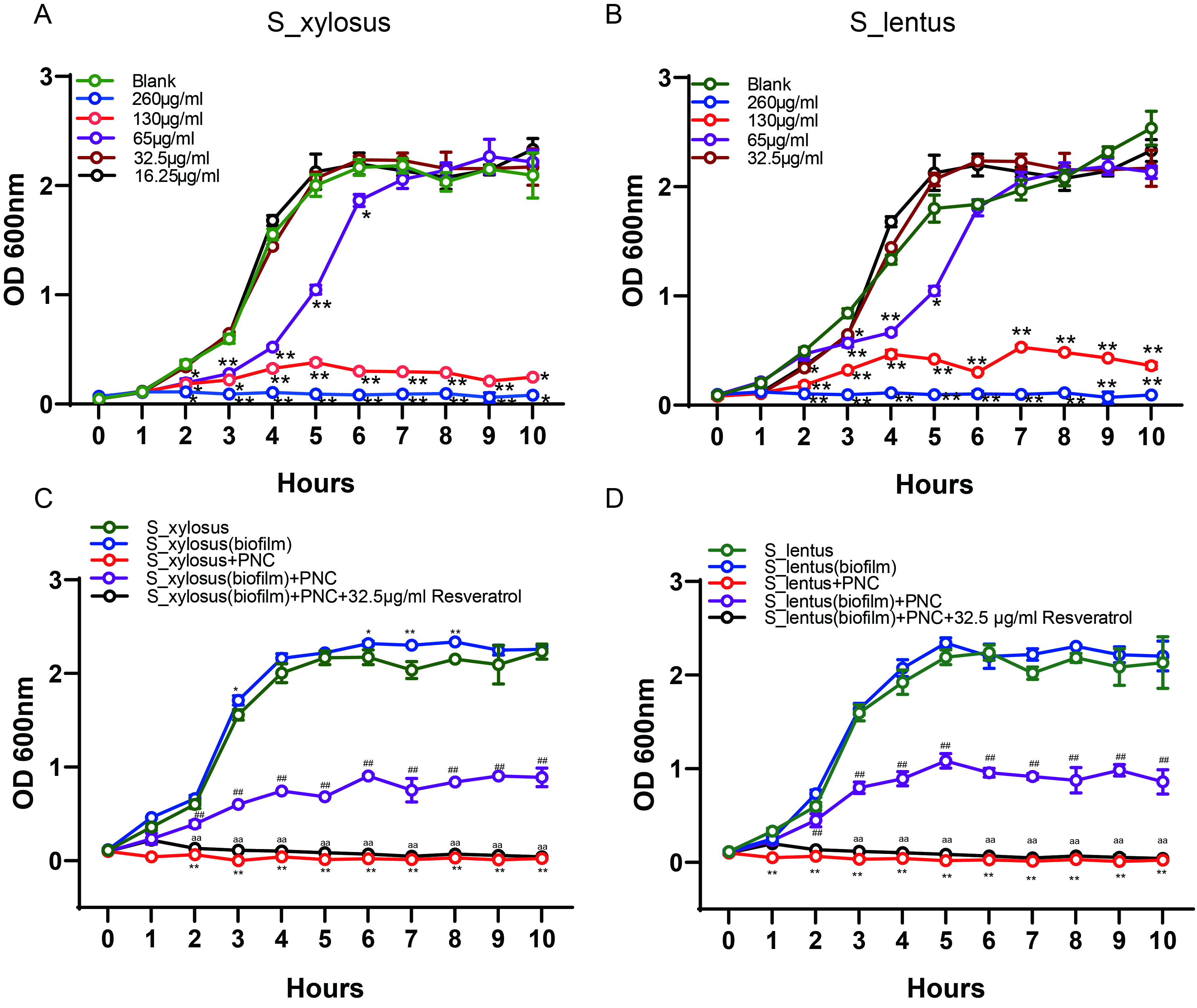

Supplement: Supplementary file 1 — Additional file 1: Figure S1. Resveratrol restrains the formation of Staphylococcus_xylosus and Staphylococcus_lentus biofilms. (A-B) Staphylococcus_xylosus and Staphylococcus_lentus were treated with resveratrol with the minimum inhibitory concentration (MIC, 260 μg/mL), 1/2 MIC, 1/4 MIC and 1/8 MIC for 0, 1, 2, 3, 4, 5, 6, 7, 8, 9, 10 h. Growth curves were applied to analyze the restraint of resveratrol on Staphylococcus_xylosus and Staphylococcus_lentus with different concentrations. (C-D) Staphylococcus_xylosus and Staphylococcus_lentus and their biofilms were treated with 1U of penicillin (PNC), and then the Staphylococcus_xylosus Staphylococcus_lentus biofilms were further treated with 1/8 MIC for 0, 1, 2, 3, 4, 5, 6, 7, 8, 9, 10 h. Growth curves were applied to analyze the synergistic effects of resveratrol and PNC on the inhibition of two types of Staphylococcus that induced biofilm formation. *P < 0.05 vs. S_xylosus or S_lentus group. **P < 0.01 vs. Blank, S_xylosus or S_lentus group, ##P < 0.01 vs. S_xylosus + PNC or S_lentus + PNC group, aaP < 0.01 vs. S_xylosus (biofilm) + PNC or S_lentus (biofilm) + PNC. Data are presented of three independent experiments and expressed as mean ± SD. [file 10020_2022_463_MOESM1_ESM.jpg]

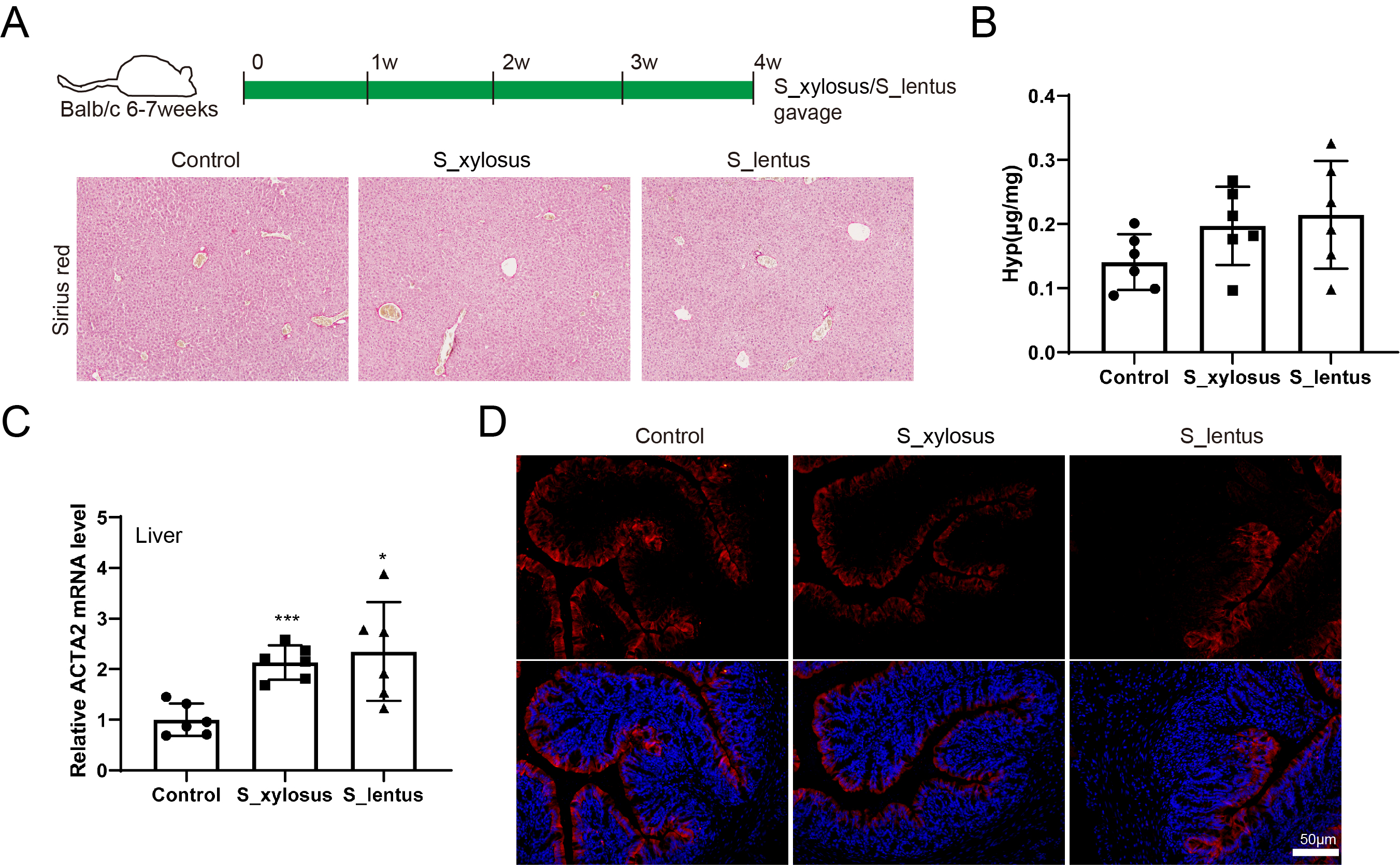

Supplement: Supplementary file 2 — Additional file 2: Figure S2. Evaluation of Staphylococcus_xylosus and Staphylococcus_lentus on liver injury in normal mice. The pure cultures of the Staphylococcus_lentus and Staphylococcus_xylosus (109 CFU) were given to normal mice by gavage. (A) The protocol for in vivo assays. The degree of liver fibrosis in mice was evaluated by Sirius red staining, one-way ANOVA with Tukey’s post-hoc. N = 5. (B) The Hyp level was detected by an ELISA kit, one-way ANOVA with Tukey's post-hoc, Mean ± SD, N = 6. (C) The mRNA level of ACTA2 (encoded α-SMA) was determined by qRT-PCR, one-way ANOVA with Tukey’s post-hoc. N = 6. (D) The relative ZO-1 positive areas were measured using immunofluorescence (scale bar = 50 µm). N = 5. *P < 0.05, ***P < 0.001 vs. Control. Data are presented of three independent assays. [file 10020_2022_463_MOESM2_ESM.jpg]

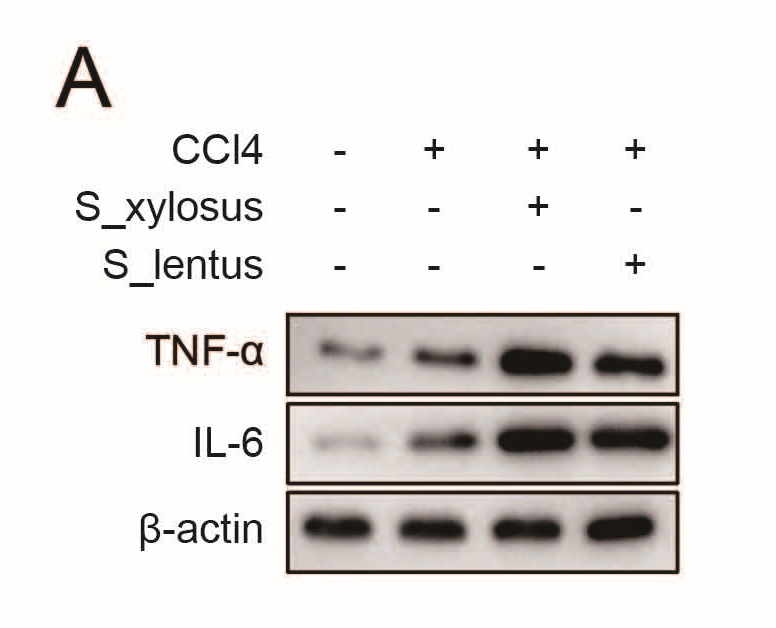

Supplement: Supplementary file 3 — Additional file 3: Figure S3. Effects of Staphylococcus_xylosus and Staphylococcus_lentus on pro-inflammatory cytokine levels in the liver of CCl4-induced mice. Mice received the pure cultures of the Staphylococcus_lentus or Staphylococcus_xylosus (109 CFU) by gavage. The intraperitoneal injection of CCl4 (0.5 µL/g) was performed to induce liver fibrosis model followed by the treatment of resveratrol (30 mg/kg) daily by gavage. The pro-inflammatory cytokine TNF-α and IL-6 in the liver was measured by western blotting. N = 6. [file 10020_2022_463_MOESM3_ESM.jpg]

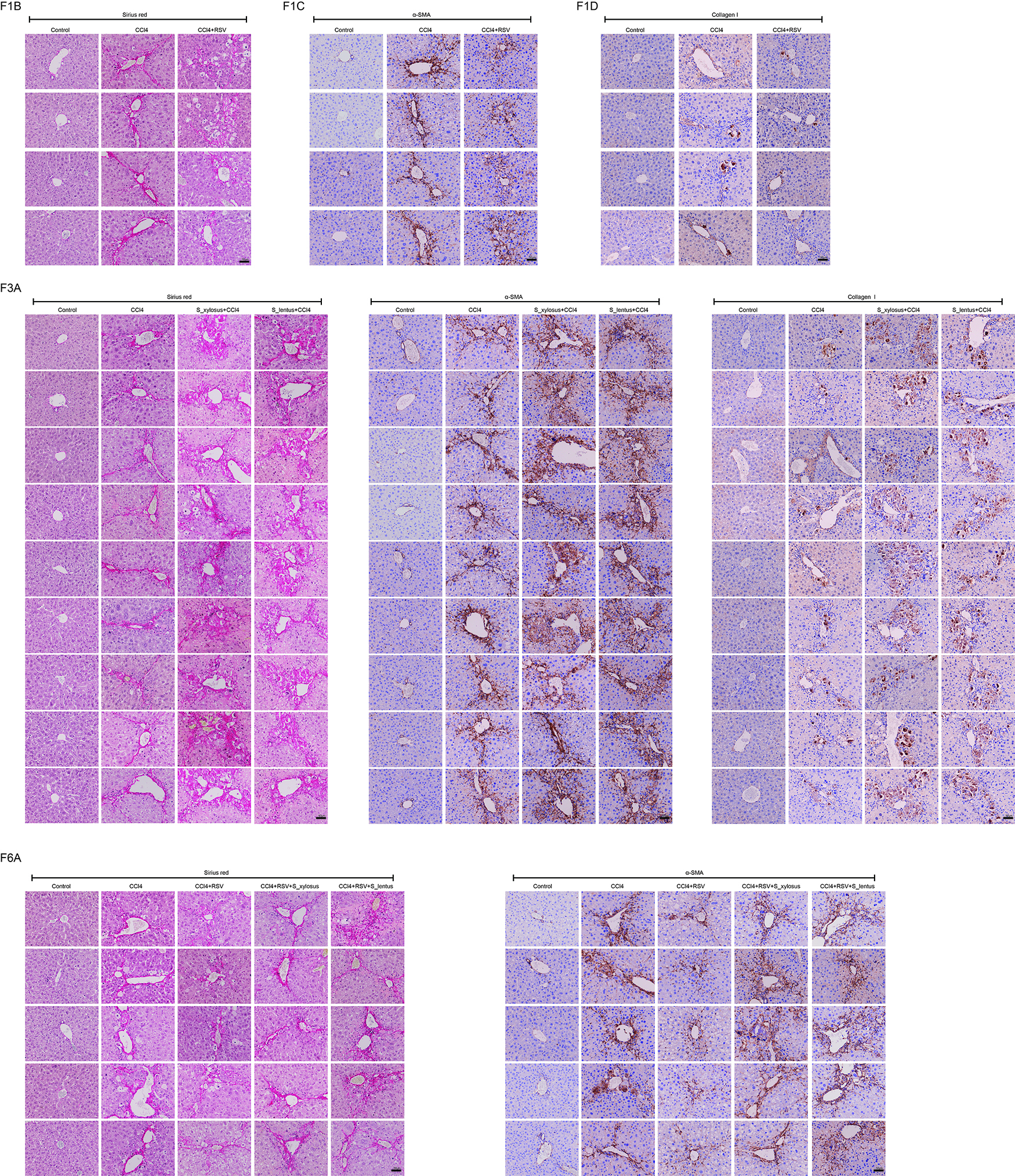

Supplement: Supplementary file 4 — Additional file 4: Figure S4. The additional representative images of Sirius red staining and immunohistochemical staining. (F1B-F1D) Sirius red staining was performed to assess the damage of liver fibrosis in mice. N = 5, scale bar = 50 µm. The expressions of α-SMA and Collagen I in mouse liver tissues were measured using immunohistochemical analysis. N = 5, scale bar = 50 µm. (F3A) Sirius red staining and immunohistochemical assays of α-SMA and Collagen I were conducted to assess liver fibrosis in mice. N = 10, scale bar = 50 µm. (F6A) Images of Sirius red staining (scale bar = 50 µm) and Immunohistochemical staining of α-SMA on mouse liver tissues. N = 6. scale bar = 50 µm. [file 10020_2022_463_MOESM4_ESM.jpg]

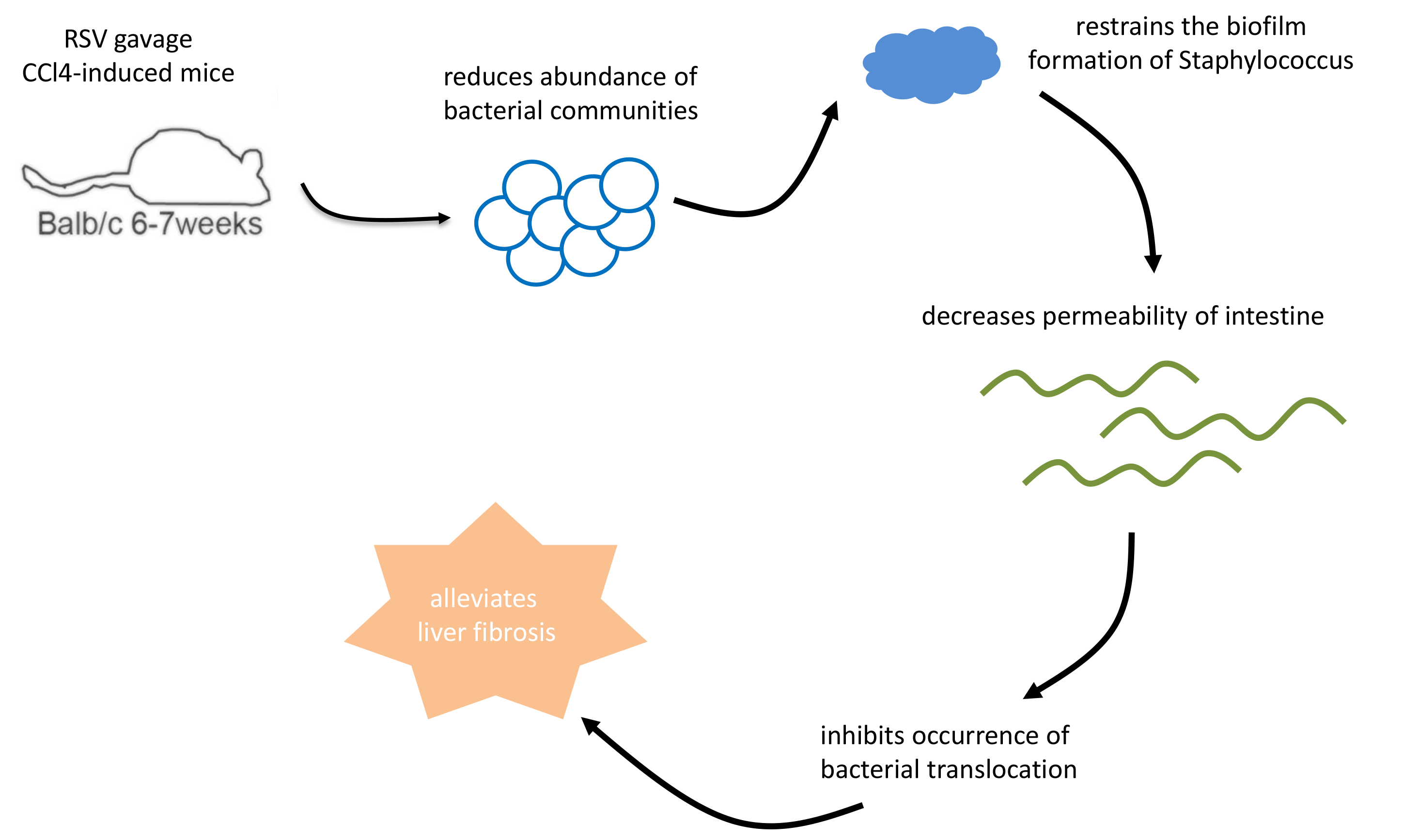

Supplement: Supplementary file 5 — Additional file 5: Figure S5. A schematic representation for resveratrol in regulating liver fibrosis. The resveratrol treatment reduced the abundance of Staphylococcus_xylosus and Staphylococcus_lentus, which were crucial for the development of liver fibrosis. Staphylococcus_xylosus and Staphylococcus_lentus promoted the occurrence of bacterial translocation via enhancing the permeability of intestine, while resveratrol treatment reversed the effect and eventually ameliorated liver fibrosis. [file 10020_2022_463_MOESM5_ESM.tif]
